# The risk of neoplasia in patients with Barrett's esophagus indefinite for dysplasia: a multicenter cohort study

**DOI:** 10.1016/j.gie.2021.01.042

**Published:** 2021-08

**Authors:** Richard Phillips, Wladyslaw Januszewicz, Nastazja D. Pilonis, Maria O'Donovan, Tarek Sawas, David A. Katzka, Rebecca C. Fitzgerald, Massimiliano di Pietro

**Affiliations:** 1 MRC Cancer Unit, University of Cambridge, Cambridge, United Kingdom; 2 Department of Gastroenterology, Hepatology and Clinical Oncology, Medical Centre for Postgraduate Education, Warsaw, Poland; 3 Department of Histopathology, Addenbrooke's Hospital, Cambridge, United Kingdom; 4 Mayo Clinic, Rochester, Minnesota, USA

**Keywords:** BE, Barrett's esophagus, BE-IND, Barrett's esophagus indefinite for dysplasia, CI, confidence interval, EAC, esophageal adenocarcinoma, FU, follow-up, HGD, high-grade dysplasia, IMC, intramucosal cancer, IQR, interquartile range, LGD, low-grade dysplasia, LSBE, long-segment Barrett's esophagus, OR, odds ratio, PPI, proton-pump inhibitor, SSBE, short-segment Barrett's esophagus

## Abstract

**Background and Aims:**

Current understanding of the risk of neoplastic progression in patients with Barrett's esophagus with indefinite dysplasia (BE-IND) stems from small retrospective and pathology registry studies. In this multicenter cohort study, we aimed to determine the incidence and prevalence of neoplasia in BE-IND.

**Methods:**

Patients with confirmed BE-IND from 2 academic centers were included if they had no previous evidence of dysplasia and underwent endoscopic follow-up (FU) of ≥1 year. The rate of progression to neoplasia was calculated and categorized as prevalent (progression within 1 year of FU) and incident (progression after 1 year of FU). Multivariable regression adjusted for relevant clinical features was performed to identify risk factors for progression.

**Results:**

Four hundred sixty-five patients diagnosed with BE-IND were identified between 1997 and 2017, of which 223 (48.0%) were excluded. Of the remaining 242 patients, 184 (76.0%) had no evidence of dysplasia during FU. In 23 patients (9.5%), prevalent neoplasia occurred (20 low-grade dysplasia [LGD], 2 high-grade dysplasia [HGD], 1 intramucosal cancer [IMC]), whereas 35 patients (14.5%) developed incident neoplasia (27 LGD, 5 HGD, 3 IMC), after a median 1.5 years (interquartile range, 0.6-3.2 years). The incidence rates of any neoplasia and HGD/IMC were 3.2 and 0.6 cases/100 patient-years, respectively. BE length correlated with an increased risk of prevalent (odds ratio, 1.18 per 1 cm; 95% confidence interval, 1.02-1.38; *P* = .033) and incident neoplasia (odds ratio, 1.02; 95% confidence interval, 1.00-1.03; *P* = .016).

**Conclusion:**

Patients with BE-IND should be closely monitored, because nearly a quarter harbor or will shortly develop dysplasia. BE length is a clinical predictor of neoplastic progression; however, more-accurate molecular biomarkers for risk stratification are warranted.

## Introduction

Barrett's esophagus (BE) occurs when squamous epithelium in the distal esophagus is replaced by a metaplastic columnar epithelium with goblet cells, which occurs in the context of chronic GERD.^1^ Compared with the general population, patients with BE are at increased risk of esophageal adenocarcinoma (EAC), which is the solid malignancy with the fastest increase in incidence in the western world.^1^^,^^2^ The malignant progression of BE is believed to occur through progressive stages, including low-grade dysplasia (LGD), high-grade dysplasia (HGD), and intramucosal cancer (IMC).^1^ Although the annual risk of malignant progression in non-dysplastic BE is estimated to be as low as 0.3%,^3^ in the presence of an LGD diagnosis, this risk increases to ∼10% per annum.^4^ Therefore, endoscopic ablation is recommended in those cases as an appropriate intervention.^5^, ^6^, ^7^, ^8^

The diagnosis of dysplasia is usually made according to the Vienna classification, which includes a 5-tier classification system based on the increasing degree of architectural and cytologic abnormalities and the presence of invasion of the neoplastic cells through the lamina propria.^9^ A diagnosis of BE with indefinite for dysplasia (BE-IND) may be given when the degree of cellular atypia is suggestive but not definite for dysplasia, which may be due to background inflammation or technical artifact. Several studies have been performed to assess the clinical behavior of BE-IND,^10^, ^11^, ^12^, ^13^, ^14^ including a recent meta-analysis.^15^ However, a substantial heterogeneity of the studies, the small sample size in most reports (n < 100), and lack of clinical data in pathology registries are significant limitations of the existing literature, which makes estimating the risk associated with BE-IND challenging.

We, therefore, conducted a multicenter cohort study analyzing prospectively collected data from 2 academic referral centers (Cambridge University and Mayo Clinic) to assess the risk of prevalent and incident neoplasia in patients with BE-IND and to identify clinical risk factors of neoplastic progression.

## Methods

### Study design

In this multicenter cohort study, we have retrospectively analyzed prospectively collected data from research databases from 2 academic referral centers in the United Kingdom (Cambridge University) and the USA (Mayo Clinic). The collected data included demographic data (age, sex, residence), clinical information (body mass index, smoking status, proton-pump inhibitor [PPI] intake), endoscopic and histologic data (the length of BE segment, stage of dysplasia, degree of background inflammation, presence of hiatus hernia), and follow-up (FU) details (date and number of FU endoscopies). The Cambridge and Mayo Clinic databases spanned from 1999 to 2018 and from 1997 to 2017, respectively. All patients provided written, informed consent to be included in the research databases, and ethics committee approval was granted Cambridge University: LREC01/149; Mayo Clinic: 9-000514. This report was written following the STROBE statement for cohort studies.^16^

### Patients

We have included consecutive adult patients (≥18 years old) with endoscopically and histologically confirmed BE (at least 1 cm of columnar-lined mucosa in the tubular esophagus with goblet-cell metaplasia), who received at least one diagnosis of BE-IND. All patients underwent endoscopic procedures with biopsies undertaken according to the Seattle protocol (gastroesophageal junction and quadrantic esophageal biopsies every 2 cm).^17^ Participating centers followed guideline recommendations for patients with BE-IND, which include optimization of PPI therapy and repeat endoscopy in 6 months (or earlier in the presence of Los Angeles grade C-D esophagitis or visible lesions). BE length, presence of visible lesions, presence and size of hiatus hernia, number and distribution of biopsies, use of PPI and change in prescription, and smoking status (never/former/active) were assessed through detailed review of endoscopy reports and electronic medical records. The following exclusion crieria were applied: (1) FU shorter than 1 year; (2) a previous diagnosis of definite dysplasia (any grade), and (3) previous history of endoscopic therapy (endoscopic resection or ablation). BE of maximum length <3 cm was considered as a short-segment BE (SSBE) and ≥3 cm as a long-segment BE (LSBE).

### Histopathology

**Figure 1:**
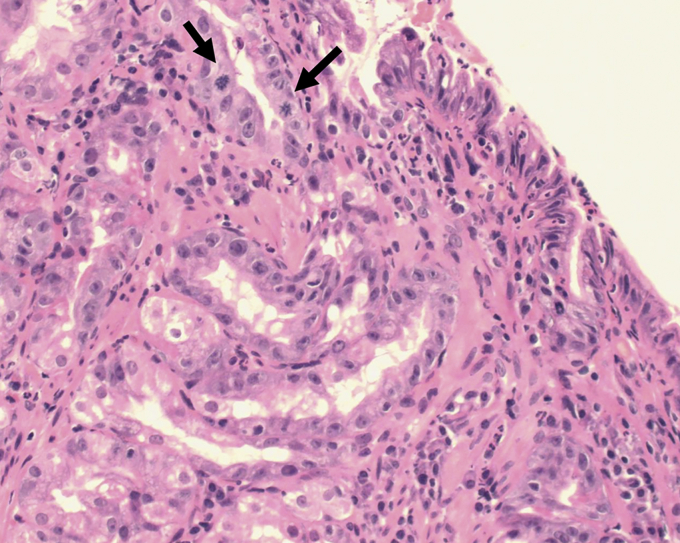
Example of Barrett’s esophagus with gastric metaplasia and indefinite for dysplasia (H&E, orig. mag. ×20). Crypt epithelium shows atypia with nuclear enlargment, some pleomorphism, membrane irregularities and mitoses (*arrows*). Surface epithelium shows degenerate changes, hyperchromasia and some stratification of nuclei with a neutrophilic infiltrate.

The histologic assessment was made using the Vienna classification.^9^ Each case of dysplasia was confirmed by a second experienced GI pathologists at each institution. When required, additional levels of the tissue blocks were performed at the discretion of the pathologist as per routine institutional practice. Histologic BE-IND diagnoses were not further reviewed for the purpose of this study, because we assumed that the original expert diagnosis was sufficiently robust. A diagnosis of BE-IND was made when there were epithelial abnormalities that were insufficient to diagnose dysplasia of any grade or the nature of the epithelial abnormalities was uncertain due to inflammation. BE-IND could also be diagnosed when there were technical factors precluding a reliable assessment of the epithelium, such as biopsy crushing artifact, thermal artifact, and tangential embedding and sectioning.^18^ An example of a case of BE-IND is presented in Figure 1.

The mucosal inflammation, both acute and chronic, at the time of the BE-IND diagnosis was assessed and categorized into (1) none, (2) mild, (3) moderate, and (4) severe. Mild inflammation was defined as very occasional neutrophils within the surface epithelium or crypt epithelium or lamina propria; moderate as the presence of crypt abscesses or scattered collections of neutrophils infiltrated within the surface epithelium; severe as associated ulceration. The highest degree of either acute or chronic inflammation was assigned to each patient.^19^

### Outcomes

The time to progression was calculated from the date of the first BE-IND diagnosis until the last available endoscopy date or the occurrence of any degree of neoplasia (other than BE-IND). Progression rates were categorized as prevalent neoplasia when the progression to any grade of neoplasia (LGD/HGD/IMC) occurred within 1 year of FU, or incident neoplasia when the progression occurred after 1 year of FU. In addition, the number of cases of BE-IND and progression rates were assessed in the 2 cohorts (Cambridge and Mayo Clinic) for cases diagnosed before and after 2007 (which divides the study timeframe evenly into 2 time periods).

### Statistics

Quantitative variables were described as means, medians with standard deviation (SD) and interquartile ranges (IQRs), where appropriate. Categorical variables were presented as counts and percentages of the cohort. The proportional event rates during FU were compared by Kaplan-Meier analysis and the log-rank test. Risk differences were calculated as the difference in the proportional event rates during FU. A multivariable regression model was used to identify risk factors for progression. For all analyses, a *P* value <.05 was considered statistically significant. The relative risk ratio for neoplasia progression was calculated using the Wald approximation of the confidence interval (CI). All analyses were performed using R Statistics version 3.4.3 (R Foundation for Statistical Computing, Vienna, Austria).

## Results

### Patients' characteristics

**Figure 2:**
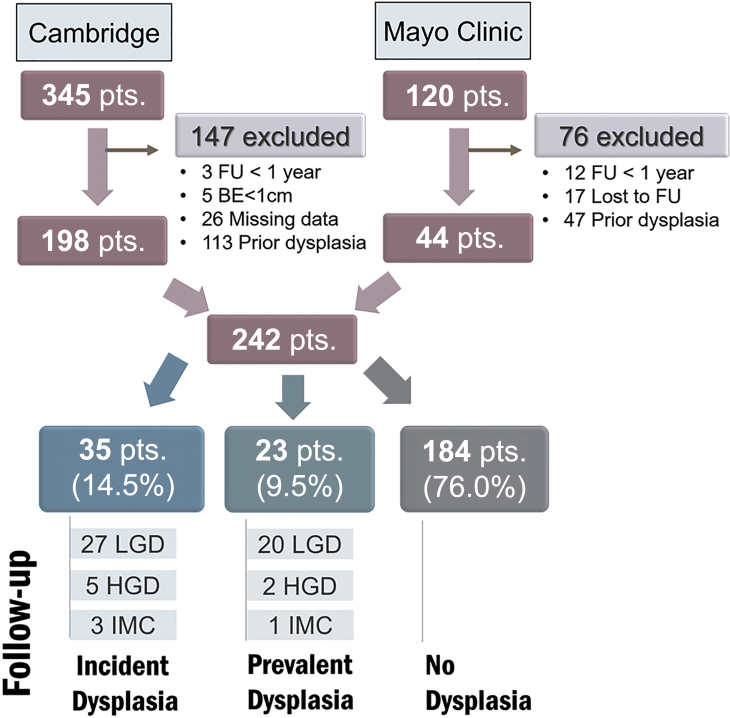
Study flowchart. *BE*, Barrett’s esophagus; *FU*, follow-up; *HGD*, high-grade dysplasia; *IMC*, intramucosal cancer; *LGD*, low-grade dysplasia; pts, patients.

A total of 465 patients with a diagnosis of BE-IND were identified (Cambridge University 345 patients [74.2%], Mayo Clinic 120 patients [25.8%]) (Figure 2). Of these, 223 patients were excluded (48.0%) due to previous history of dysplasia (n = 160), FU <1 year (n = 15), being lost to FU (n = 17), missing clinical/endoscopy data (n = 26), or BE <1 cm (n = 5). The remaining 242 patients (52.0%) were included in the analysis (mean age, 63.7 years [±11.6 years], 178 males [73.6%]). Patients characteristics are shown in Table 1. The median FU time was 6.5 years (interquartile range [IQR] 2.7-12.6 years). At the time of first BE-IND diagnosis, 29 patients (12.0%) had no features of background inflammation, 108 (44.6%) had mild inflammation, 79 (32.6%) had moderate inflammation, and 6 (2.5%) had severe inflammation. In most cases, the diagnosis of BE-IND was made due to background inflammation (n = 186; 76.9%) and less often due to cellular atypia of unknown significance (n = 43; 17.8%) or technical artifact (n = 7; 2.9%). The BE-IND diagnosis was established by targeted biopsies of a mucosal abnormality in a quarter of cases (n = 61; 25.2%), and in the remaining cases, it was diagnosed in nontargeted biopsies from the BE segment (n = 179 cases; 74.0%). Strict adherance to Seattle protocol biopsies was achieved in 70% of cases (n = 170).

Most of the patients (202 of 242; 83.5%) were on PPIs at the time of the BE-IND diagnosis. Most of the patients had their PPI dose increased after the BE-IND diagnosis (138 of 242; 57.0%), whereas in about a third (76 of 242; 31.4%) the treatment dose remained unchanged; 28 patients (11.6%) had missing data on PPI therapy. The median time to first FU endoscopy after BE-IND diagnosis was 6.7 months (IQR, 5.4-10.1).

### Outcomes

**Figure 3:**
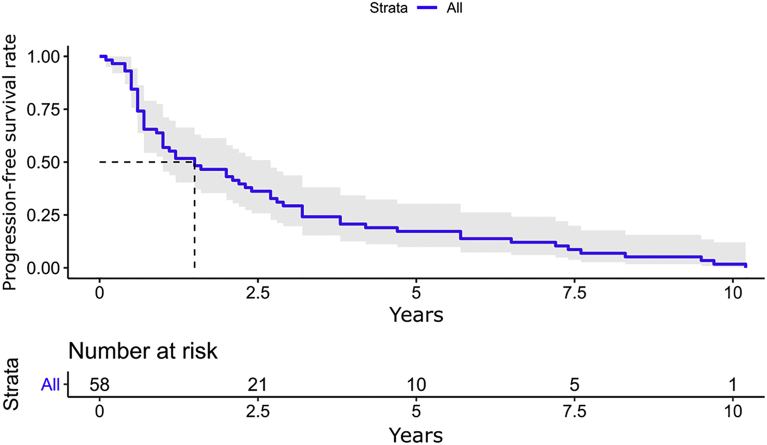
Time to progression (both prevalent and incident neoplasia).

Overall, 58 patients (24.0%) developed dysplasia during the FU (23 prevalent [9.5%] and 35 incident [14.5%]) (Figure 2). The median time to the diagnosis of neoplasia (both prevalent and incident) was 1.5 years (IQR, 0.6-3.2 years), as shown in Figure 3. The detection rates of any neoplasia, HGD/IMC, and cancer were 3.2, 0.6, and 0.2 per 100 patient-years, respectively. When looking only at cases with incident neoplasia (n = 35), the median time to progression was 2.9 years (IQR, 2.0-5.7 years). When comparing the Mayo Clinic and Cambridge cohorts, there was no difference in detection rates (log-rank *P* = .5).

### Risk factors for neoplasia progression

We performed a multivariate logistic regression analysis adjusted for age, sex, BE length, the extent of IND (focal vs multifocal), persistent IND (yes vs no), degree of inflammation (none/mild/moderate/severe), and smoking status to identify factors associated with neoplastic progression (Supplementary Table 2, Supplementary Table 3, Supplementary Table 4, available online at www.giejournal.org).

First, we have looked at whether the degree of background mucosal inflammation had an influence on the progression of BE-IND cases. Of those patients who progressed to neoplasia, 8 had no inflammation (13.8%), 26 had mild inflammation (44.8%), and 20 had moderate inflammation (34.5%). None of the patients who progressed had severe background inflammation at the time of the initial BE-IND diagnosis. The degree of background inflammation was not associated with the development of dysplasia in the multiavriable regression model (both prevalent [*P* = .765] and incident [*P* = 0.451]; Supplementary Table 2, Supplementary Table 3, Supplementary Table 4, available online at www.giejournal.org).

**Figure 4:**
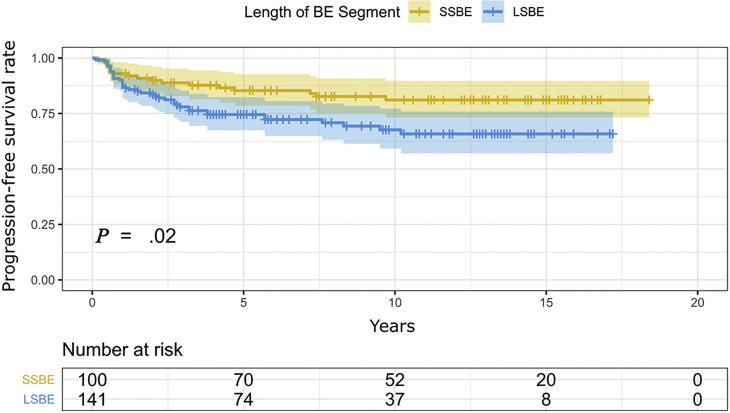
Prevalent and incident neoplasia risk in long- and short-segment Barrett’s esophagus (BE). *LSBE*, Long-segment Barrett’s esophagus; *SSBE*, short-segment Barrett’s esophagus.

The length of the BE segment was the only significant risk factor for neoplastic progression (odds ratio [OR], 1.21 for every 1 cm of BE segment; 95% CI, 1.08-1.37; *P* = .001; Supplementary Table 2, available online at www.giejournal.org). When looking separately at the prevalent and incident cases, similarly, BE length remained a relevant risk factor with an OR of 1.18 (95% CI, 1.02-1.38; *P* = .033) and OR of 1.02 (95% CI, 1.00-1.03; *P* = .016) for every 1 cm of BE, respectively (Supplementary Tables 3 and 4, available online at www.giejournal.org). Analogously, the detection rates were significantly higher for LSBE than for SSBE (log-rank *P* = .021), as shown in Figure 4. The relative risk for neoplastic progression in LSBE (vs SSBE) was 1.7 (95% CI, 1.01-2.77).

### Timeline of diagnosis and rates of progression

To analyze the trends in BE-IND diagnoses within institutions and how the progression rates changed over time, we divided the cohort from the 2 centers based on the year of diagnosis before and after 2007. In the Cambridge and Mayo Clinic cohorts, 113 (57.1%) and 16 (36.4%) BE-IND cases were diagnosed before 2007, and 85 (42.9%) and 28 (63.6%) after 2007, respectively. The progression rates (to both prevalent and incident neoplasia) were numerically higher in patients diagnosed after 2007, compared with those diagnosed before 2007 (4.8 vs 2.6 cases per 100 patient-years, respectively, *P* = .800). Looking at an institutional level, this difference was statistically significant in the Mayo Clinic cohort (10.8 vs 1.9 cases per 100 patient-years, *P* = .02) and numerically higher in the Cambridge cohort (3.6 vs 2.6 cases per 100 patient-years, *P* = .400).

## Discussion

In this multicenter cohort study, we have shown that a substantial proportion of patients with BE-IND harbored prevalent neoplasia diagnosed at early endoscopic FU (∼10%), and a higher proportion of patients (∼15%) developed incident neoplasia during the FU. The risk of neoplastic progression in BE-IND is higher than in nondysplastic BE; therefore, this diagnosis warrants close endoscopic monitoring. The length of the BE segment was the only clinical factor correlated with the increased risk of development of both prevalent and incident neoplasia, with LSBE associated with over a 1.7-fold increase in this risk compared with SSBE.

The incidence rate of any neoplasia (LGD/HGD/IMC) in our study yielded 3.2 cases per 100 patient-years. This number falls within values previously reported in smaller retrospective cohort studies showing an incidence ranging from 1.4 to 5.9 cases per 100 patient-years.^20^^,^^21^ Interestingly, the incidence rate of HGD/IMC (0.6 cases/100 patient-years) was lower than in previous reports, including a recent meta-analysis (1.5 cases/100 patient-years)^15^ and previous smaller retrospective cohort studies^11^^,^^13^^,^^14^^,^^20^^,^^21^ (Supplementary Table 1, available online at www.giejournal.org). High compliance with PPI treatment (83.5%) and timely endoscopic ablation in our cohort may have contributed to a lower rate of progression to more advanced BE stages.

In terms of the management of BE-IND, there is consensus among European and American guidelines on recommending repeat endoscopy in 6 months and optimization of acid suppressant medication treatment, whereas endoscopic ablation is not recommended.^5^^,^^7^^,^^8^ Our data confirm high compliance with the guideline recommendations in this study cohort. In particular, the BE-IND diagnosis was followed by an increase in PPI dosage in most cases (57.0%), and the median time to first FU endoscopy was 6.7 months (IQR, 5.4-10.1).

In our study, over 30% of patients had evidence of persistent IND; however, this was not identified as a risk factor for progression in a multivariate regression model. The length of the BE segment was the only identifiable risk factor for both prevalent and incident neoplasia, which is in agreement with previous retrospective cohort studies.^12^^,^^13^^,^^20^ Given that both BE-IND and length of BE are factors associated with increased risk of progression, this study suggests that patients with long segments of BE and a BE-IND diagnosis should be more closely monitored, even when the FU endoscopy shows no definite dysplasia. Of interest, European Society of Gastrointestinal Endoscopy recommends that all patients with BE longer than 10 cm should be referred to expert centers.^5^ Moreover, the high rate of prevalent neoplasia at first FU support lowering the threshold for endoscopic resection of subtle lesions within BE segments with a previous BE-IND diagnosis to provide a larger tissue specimen for analysis.

BE-IND remains a subjective diagnosis, and the threshold for this pathologic diagnosis might have changed during the study period. This is likely to have occurred because, in the last decade, pathologists have become increasingly aware of the issues related to overdiagnosis of dysplasia, in particular LGD.^4^^,^^22^ As the threshold for definite dysplasia diagnosis increases, cases with subtle dysplastic changes can be diagnoses as BE-IND, with potential impact on progression risk. Although this hypothesis is difficult to demonstrate within this study, we did observe higher rates of neoplastic progression in patients diagnosed with BE-IND after 2007. This finding may suggest that the BE-IND diagnosis has become more clinically significant over time. It also supports the requirement of a second GI pathologist to corroborate this complex diagnosis.

Although background inflammation was the most common reason driving the BE-IND diagnosis, we did not find a correlation between the degree of inflammation and the progression rate. Likely, the current classification of inflammation (acute/chronic, mild/moderate/severe) does not accurately quantify the inflammatory burden and the related risk of progression. It was shown that a gradient in pro-inflammatory interleukins (IL-8 and IL-1β) exists along the length of the BE segment, and this is responsive to acid and bile salt exposure.^23^ Likewise, in a mouse model of esophageal carcinogenesis, IL-1β overexpression induced adenocarcinoma via activation of the NOTCH pathway.^24^ Therefore, more accurate biomarkers of inflammation are likely required to quantify the inflammatory burden in a more meaningful way.

So far, research into molecular biomarkers has provided significant insight into the possibility of predicting disease behavior, although this has not yet modified clinical practice. EAC is highly heterogeneous at the molecular level, and identifying a single test to risk stratify patients has been a challenge.^25^ Assessment of p53 protein expression by immunohistochemistry is promising, because the mutation of the *TP53* gene is the most recurrent event in esophageal carcinogenesis. Kastelein et al^26^ performed a large case-control study and showed that aberrant p53 expression by immunohistochemistry was associated with an increased risk of progression with a relative risk of 5.6 (95% CI, 3.1-10.3). In a recent multicenter prospective cohort study, we found that aberrant p53 expression correlated with short-term neoplastic progression, suggesting the presence of histologically missed prevalent dysplasia at the time of a negative endoscopy. In keeping with the evidence that EAC has a high frequency of gross DNA aberrations, cytometric detection of DNA aneuploidy correlated with increased risk of long-term neoplastic progression in BE.^27^^,^^28^ We have recently devised a simple and clinically applicable methodology to assess DNA content aberrations by shallow whole-genome sequencing, which can find aberrant genomic signals as early as 10 years before histopathologic progression.^29^ This opens exciting venues for prediction of disease behavior in BE and, in particular, BE-IND.

The strengths of this multicenter study are the size of the cohort of BE-IND patients and the robust histopathologic diagnosis by experienced pathologists. The diagnosis of BE-IND is generally affected by significant interobserver variability, and lack of expert diagnosis can have an impact on the rate of progression. Many previous cohort studies in the field of BE-IND failed to recognize the heterogeneity between BE-IND patients, which arises due to the complexities and subjectivity of the diagnosis.^30^

This study also has several limitations. First, although clinical data were prospectively collected, the analysis of the BE-IND cohort was conducted retrospectively, with a degree of missing data. Second, it is known that a BE-IND diagnosis is associated with poor intraobserver agreement between histopathologists (ҡ =.18).^10^ Although we did not use a central panel for confirming the historical diagnosis, expert pathologists from the original institutions double reported the cases. This may have introduced a selection bias that makes these results difficult to generalize to nonexpert centers. Furthermore, p53 protein expression assessment may help improve the agreement and reproducibility of the dysplasia diagnosis among pathologists.^31^, ^32^, ^33^ However, we could not perform p53 immunohistochemistry on all of our cases because many were historical. The design of this study relied on standard hematoxylin and eosin staining in combination with other clinical and endoscopic data. Finally, there were insufficient data to investigate the chemopreventive role of PPIs and other pharmacological interventions (such as nonsteroidal antiinflammatory drugs and statins) due to the lack of adequate information. Although we found no correlation between the severity of inflammation and the rate of progression, we could not establish whether optimization of PPI therapy had an impact on this risk.

In conclusion, the BE-IND diagnosis represents a difficult histopathologic diagnosis, but it does correlate with an increased risk of both prevalent and incident neoplasia; therefore, early endoscopic FU remains the mainstay. The length of the BE segment was the only identifiable clinical risk factor for neoplastic progression, and no other clinical variable was useful in quantifying this risk. In the future, more accurate risk stratification tools will be required, including the use of molecular biomarkers.

**Table 1:** Patient characteristics

Characteristic	Value
Cohort	242
Cambridge University	198 (81.8)
Mayo Clinic	44 (18.2)
Male sex	178 (73.6)
Age (years), mean (±SD)	63.7 (±11.6)
PPI intake (at the time of initial diagnosis)	202 (83.5)
Multifocal IND	50 (20.7)
Persistent IND	74 (30.6)
Barrett’s length (cm), mean (±SD)∗	
Circumferential	2.6 (±3.8)
Maximum	5.0 (±3.6)
Presence of hiatus hernia	125 (51.7)
Smoking status	
Never	101 (41.7)
Former	61 (25.2)
Active	11 (4.5)
Unknown	69 (28.5)
Inflammation status†	
None	29 (12.0)
Mild	108 (44.6)
Moderate	79 (32.6)
Severe	6 (2.5)
Missing data	20 (8.3)
Cause of the BE-IND diagnosis	
Inflammation	186 (76.9)
Cellular atypia of unknown significance	43 (17.8)
Technical artifact	7 (2.9)
Unknown	6 (2.5)

Values are number (%) except where indicated otherwise.

*IND*, Indefinite for dysplasia.

∗ One patient had missing data on the length of the Barrett’s segment.

† Inflammation at the time of Barrett’s esophagus with indefinite dysplasia (BE-IND) diagnosis. In cases of persistent BE-IND, the highest grade of inflammation was presented.

## Figures and Tables

**Supplementary Table 1:** Available studies on Barrett’s indefinite for dysplasia

Study	Country	Years	Study type	Data source	No. of BE-IND cases	Any neoplasia rate (LGD/HGD/EAC)	Advanced neoplasia rate (HGD/EAC)	Risk factors for incident neoplasia
Ma et al, 2017^20^	USA	2000-2014	Retrospective cohort	Pathology database + clinical database	106	1.4 cases/100 person-years	0.9 cases/100 person-years	Smoking, BE length
Horvath et al, 2015^13^	USA	1992-2007	Retrospective cohort	Pathology database	107	4.5 cases/100 person-years	1.2 cases/100 person-years	BE length, multifocal IND
Sinh et al, 2015^14^	USA	NA	Retrospective cohort	BEST database (USA)	83	NA	0.86%/year	None
Kestens et al, 2015^11^	the Netherlands	2002-2011	Retrospective cohort	Pathology database (PALGA)	842	NA	1.4 cases/100 person-years	Advancing age (per 10 years)
Sonwalkar et al, 2010^10^	UK	1984-2004	Retrospective cohort	Histology database	41	NA	NA	None
Choi et al, 2015^12^	USA	Mixed	Meta-analysis (including Sonwalkar et al,^10^ Kestens et al,^11^ Sinh et al,^14^ Horvath et al^13^)	Multiple histology databases	1176	NA	0.43-1.2 cases/100 person-years	BE length, multifocal IND, advancing age (>60 years), abnormal p53 expression
Krishnamoorthi et al, 2020^15^	USA	Mixed	Meta-analysis	Multiple histology databases	1441	NA	1.5 cases/100 person-years	NA
Henn et al, 2020^21^	USA	2006-2016	Retrospective cohort study		107	5.95 per 100 person-years	0 cases per 100 person-years	Persistent IND (OR, 3.23; 95% CI, 1.04-9.98)

*BE-IND*, Barrett’s esophagus indefinite for dysplasia; *LGD*, low-grade dysplasia; *HGD*, high-grade dysplasia; *EAC*, esophageal adenocarcinoma; *NA*, not available; *OR*, odds ratio; *CI*, confidence interval.

**Supplementary Table 2:** Multivariable linear regression model results for predictors of any neoplastic progression (both prevalent and incident) in Barrett’s esophagus indefinite for dysplasia

Variable	Coefficient	95% Confidence interval	*P* value
Gender	1.39	0.49-4.30	.542
Age	0.97	0.93-1.01	.170
Inflammation status∗	1.24	0.71-2.21	.448
BE length (max cm)	1.21	1.08-1.37	.001
Multifocal BE-IND† (yes vs no)	1.22	0.40-3.59	.719
Persistent BE-IND‡ (yes vs no)	1.07	0.41-2.75	.893
Body mass index	1.05	0.97-1.15	.190
Smoking (ever vs never)	1.34	0.54-3.36	.527

*P* values <.05 are significant.

*BE*, Barrett’s esophagus; *BE-IND*, Barrett’s esophagus indefinite for dysplasia.

∗ Inflammation status was included as ordinal data and categorized as 0- no inflammation, 1- mild, 2-moderate, and 3- severe inflammation.

† Multifocal BE-IND was categorized as present (1) when more than 1 biopsy within a single Barrett's segment at the index endoscopy showed indefinite for dysplasia mucosal changes.

‡ Persistent BE-IND was categorized as present (1) when a single patient recieved more than 1 diagnosis of Barrett’s indefinite for dysplasia with in the follow-up period.

**Supplementary Table 3:** Multivariable linear regression model results for predictors of prevalent neoplasia in Barrett’s esophagus indefinite for dysplasia

Variable	Coefficient	95% Confidence interval	*P* value
Gender	2.81	0.61-18.8	.226
Age	1.02	0.96-1.08	.488
Inflammation status∗	1.13	0.50-2.47	.765
BE length (max cm)	1.18	1.02-1.38	.033
Multifocal BE-IND† (yes vs no)	5.62	1.4-25.63	.018
Persistent BE-IND‡ (yes vs no)	0.32	0.06-1.34	.152
Body mass index	1.13	1.02-1.27	.021
Smoking (ever vs never)	1.50	0.42-5.57	.528

*P* values <.05 are significant.

*BE-IND*, Barrett’s esophagus indefinite for dysplasia.

∗ Inflammation status was included as ordinal data and categorized as 0- no inflammation, 1- mild, 2-moderate, and 3- severe inflammation.

† Multifocal BE-IND was categorized as present (1) when more than 1 biopsy within a single Barrett's segment at the index endoscopy showed indefinite for dysplasia mucosal changes.

‡ Persistent BE-IND was categorized as present (1) when a single patient recieved more than 1 diagnosis of Barrett’s indefinite for dysplasia within the follow-up period.

**Supplementary Table 4:** Multivariable linear regression model results for predictors of incident neoplasia in Barrett’s esophagus indefinite for dysplasia

Variable	Coefficient	95% Confidence interval	*P* value
Gender	0.98	0.86-1.12	.760
Age	0.99	0.99-1.00	.022
Inflammation status∗	1.03	0.95-1.11	.451
BE length (max cm)	1.02	1.00-1.03	.016
Multifocal BE-IND† (yes vs no)	0.86	0.74-0.99	.038
Persistent BE-IND‡ (yes vs no)	1.09	0.97-1.24	.151
Body mass index	1.00	0.99-1.01	.557
Smoking (ever vs never)	1.01	0.90-1.14	.818

*P* values <.05 are significant.

*BE-IND*, Barrett’s esophagus indefinite for dysplasia.

∗ Inflammation status was included as ordinal data and categorized as 0- no inflammation, 1- mild, 2-moderate, and 3- severe inflammation.

† multifocal BE-IND was categorized as present (1) when more than 1 biopsy within a single Barrett's segment at the index endoscopy showed indefinite for dysplasia mucosal changes.

‡ Persistent BE-IND was categorized as present (1) when a single patient recieved more than 1 diagnosis of Barrett’s indefinite for dysplasia within the follow-up period.
